# Factors influencing risky single occasion drinking in Canada and policy implications

**DOI:** 10.1186/s13690-017-0190-z

**Published:** 2017-05-15

**Authors:** Ellen Rafferty, William Ian Andrew Bonner, Jillian Code, Keely McBride, Mustafa Andkhoie, Richa Tikoo, Stephanie McClean, Colleen Dell, Michael Szafron, Marwa Farag

**Affiliations:** 1 School of Public Health, University of Saskatchewan, 104 Clinic Place, Saskatoon, SK S7N 2Z4 Canada; 2 Department of Sociology, University of Saskatchewan, 1019 - 9 Campus Drive, Saskatoon, SK S7N 5A5 Canada

**Keywords:** Risky single occasion drinking RSOD in Canada, Health policy, Stress, Smoking, Alcohol, Education, Social determinants, Provinces

## Abstract

**Background:**

Misuse of alcohol, including single risky occasion drinking (RSOD) is associated with a number of health, social and economic consequences. While research demonstrates that many factors contribute to individuals’ drinking practices, little is known about risk factors that contribute to RSOD in the Canadian population. The objectives of this study are to examine the patterns of RSOD in Canada, to identify factors associated with RSOD, and to explore policy implications.

**Methods:**

The Canadian Community Health Survey (CCHS) 2009–2010 annual component was used to conduct all the analyses in this paper. We used two models: (1) a binary logistic regression model, and (2) a multinomial logistic regression model, to identify factors that were significantly associated with our dependent variables, RSOD engagement and frequency of RSOD, respectively.

**Results:**

Daily smokers were 6.20 times more likely to engage in frequent RSOD than those who never smoke. Males were 4.69 times more likely to engage in risky RSOD. We also found significant associations between the frequency of RSOD and Province/Territory of residence, income and education, marital status and perceived health status. Finally, stress was associated with engaging in infrequent RSOD.

**Conclusions:**

Our finding associating daily smoking with risk alcohol intake specifically suggests the possibility of combining public health interventions for both. The study findings also indicate that education is a protective factor, further supporting the role of education as a major determinant of health. The significant provincial variation we found also point to the need to study this issue further and understand the links between provincial level policies and RSOD.

## Background

From a public health perspective, alcohol consumption is a challenging issue to address. Controversy existed in the research community whether low levels of alcohol consumption have protective effects, and if these effects outweigh known harms; however, there is a growing consensus in the literature that the positive effects of alcohol consumption have been overestimated in the past [1–4]. Defining low-risk consumption has proved methodologically difficult, resulting in a variety of guidelines for low-risk alcohol intake across countries [5]. Likewise, alcohol misuse represents a wide spectrum of terms, from exceeding low-risk guidelines to alcohol dependence. A glossary of terms used in this article is outlined in Table 1.

**Table 1:** Glossary of Terms Associated with Alcohol Consumption

Alcohol abuse	Is a pattern of drinking that results in harm to one’s health, interpersonal relationships, or ability to work [62].
Alcohol dependency	Dependency on alcohol, also known as alcohol addiction and alcoholism, is a chronic disease. The signs and symptoms for alcohol dependence include a strong craving for alcohol, continued use despite repeated physical, psychological, or interpersonal problems and the inability to limit drinking [62].
Binge drinking	A pattern of alcohol consumption that brings the blood alcohol concentration level to 0.08% or more. This pattern of drinking usually corresponds to 5 or more drinks on a single occasion for men or 4 or more drinks on a single occasion for women, generally within about 2 h [26].
Excessive alcohol use	Excessive drinking, or excessive alcohol use includes binge drinking, heavy drinking, any alcohol use by people under the minimum legal drinking age, and any alcohol use by pregnant women [63].
Heavy drinking	For men heavy drinking is typically defined as consuming 15 drinks or more per week. For women, heavy drinking is typically defined as consuming 8 drinks or more per week [63].
Heavy episodic drinking	Defined by the World Health Organization (WHO) as 60 or more grams of pure alcohol on at least one single occasion at least monthly [15].
Risky drinking	Women having more than 3 drinks or men having more than 4 drinks on any single occasion once per month or more often [64].
Risky single occasion drinking (RSOD)	Having X number of standard drinks or more (X+) on one occasion. This definition may vary across countries by number of drinks as well as grams of alcohol per drink [65]. This article uses 5 or more drinks regardless of sex to define RSOD, based on the CCHS classification, which is equivalent to consuming 70 g or more of pure alcohol on one single occasion.

Despite variation in how different organizations define and convey low-risk drinking, it is clear that the misuse of alcohol, including risky single occasion drinking (RSOD), is associated with a number of negative health, social, and economic consequences [6, 7]. Direct health implications associated with alcohol misuse include dependency, liver cirrhosis, organ damage, diabetes, cardiovascular disease, and various types of cancer [7, 8]. Furthermore, impaired judgement, impaired driving ([9]), injury, suicide, and risky sexual behaviour may be prompted by high levels of alcohol consumption, suggesting there may be broader health and social repercussions [7, 10]. In Canada, the most recent comprehensive economic analysis of alcohol-related costs was conducted in 2006 using data from 2002, and estimated that a total of CAD 14.6 billion was spent that year on direct and indirect costs [11, 12]. These costs are associated with healthcare, law enforcement, and productivity losses [7, 12]. Direct healthcare costs alone accounted for CAD 3.3 billion in spending in 2002 [7, 12]. More recently in 2013, the costs of Fetal Alcohol Spectrum Disorder (FASD) in Canada alone totalled approximately CAD 1.8 billion dollars [13] In contrast, the societal and healthcare costs of excessive alcohol use in the United States have been estimated at USD 223.5 billion and 24.5 billion, respectively [14]. Worldwide, alcohol abuse accounted for approximately 3.3 million deaths and 139 million disability-adjusted life years (DALYS) due to injury and morbidity [15]. Inhabitants of the North American region drank 8.4 l of pure alcohol per capita in 2010, 35% higher than the world average (6.2L) and second only to the European region (10.9 L). Among current alcohol drinkers in the Americas, the prevalence of heavy episodic drinking is 22%, again second only to Europe (22.9%). In the United States, the prevalence of monthly binge drinking defined in the article as 5+ drinks for men, 4+ for women in one occasion is approximately 17% [16].

A substantial proportion of these alcohol-related harms are associated with populations who exceeded low-risk alcohol guidelines, which is determined by quantity and/or frequency of alcohol use [7, 14, 17]. As the frequency of RSOD increases, the likelihood of negative health and social consequences increases [7, 17]. In addition, there is some evidence from the United States which indicates that moderate drinkers contributed to the majority of RSOD episodes ([18]).

Research shows that a number of economic, cultural, and historical factors contribute to individuals’ drinking practices [1, 19]. Data from the National Epidemiologic Survey on Alcohol and Related Conditions (NESARC) and the National Survey on Drug Use and Health (NSDUH) demonstrated associations between early initiation of drinking (before the age of 21), age of 20–29 years, enrollment in college, being male and an increased engagement in RSOD, [1, 20]. However, it appears the gender gap is decreasing among younger cohorts [8]. In general, the factors that impact alcohol consumption have not been well explored within a Canadian context. Of particular interest may be how location of residence influences alcohol consumption in Canada, as provincial and territorial (P/T) jurisdiction over alcohol policy has created a patchwork of liquor regulations throughout the country [19, 21]. Studying provincial variations in alcohol consumption may provide insight into the impact of public policy, along with other cultural and socio-political differences, on RSOD. Furthermore, a deeper understanding of the risk factors that contribute to RSOD with respect to P/T may help guide relevant and informed alcohol related policies for each P/T.

Although experts agree that alcohol consumption can lead to negative health, social and economic consequences, defining low-risk intake has proved challenging for researchers and organizations [5, 12, 22]. Both volume and patterns of alcohol use can lead to separate risks, and studies have varied in their assessment of risk, with some focused on dose–response curves, while others focused on alcohol-attributable fractions [5]. Grams of pure alcohol per standard drink varies widely worldwide and portion sizes served often differ from the standard drink [5]. These debates added to the complexity for P/T governments and non-profit organisations to determine Canada’s Low Risk Alcohol Drinking Guidelines and communicate them to the public. In 2011, the National Alcohol Strategy Working Group (NASWG) recommended that, based on the available evidence, no more than two drinks should be consumed on most days for women (28 g of ethanol) and no more than three a day for men (42 g of ethanol) [23–25]. Similarly, the guidelines recommend that over the duration of a week, women should consume less than 10 drinks and men less than 15 (140 and 210 g of pure alcohol, respectively) [23]. The CCSA defines risky drinking as “women having more than 3 drinks or for men more than 4 drinks on any single occasion once per month or more often” [23]. In contrast, the Canadian guidelines surrounding the definition of low-risk drinking are stricter compared to guidelines in the United States. For women, the National Institute on Abuse and Alcoholism (NIAA) [26] decrees that low-risk drinking consists of no more than 3 drinks per day and 7 per week (42 and 98 g of pure alcohol, respectively), whereas for men the guideline is 4 and 14 respectively (56 and 196 g of pure alcohol, respectively) [26]. European guidelines vary widely by country, and in response to this, recent practice principles from the Joint Action Reducing Alcohol Related Harm (RARHA) in Europe and the Center for Addiction and Mental Health (CAMH) have attempted to identify a standard definition of risk. They indicate that a level of alcohol intake of 10 g or less per day would result in an alcohol attributed death rate of below 1 in 100, and provide this as a suggested maximum for low risk drinking [27, 28]. This would equate with less than one standard drink per day in Canada, where one standard drink contains approximately 14 g of pure alcohol [24, 25].

The overall purpose of this study is to explore the past-year prevalence of RSOD in Canada and the factors associated with any RSOD and frequency of RSOD in Canada. Understanding the underlying factors contributing to RSOD in Canada will help guide the development of more effective policy interventions to curtail high-risk consumption. The two research questions developed were:

1. What factors determine the likelihood of individuals engaging in any RSOD over the past 12 months?
2. What factors determine the likelihood of engaging in frequent RSOD?

We defined infrequent RSOD as (past-year occurrence of drinking 5+ drinks (70+ grams alcohol) once a month or less) and frequent RSOD as (past-year occurrence of drinking 5+ drinks (70+ grams alcohol) more than once a month).

## Methods

The Canadian Community Health Survey (CCHS) 2009–2010 annual component was used to conduct all the analyses in this paper ([29]). The sample size of this survey was 124,188. The response rate for the outcome variable of interest “How often in the past 12 months have you had 5 or more drinks on one occasion?” was 74.3%. Since logistic regression modeling uses listwise exclusion of missing values, the sample size of the regression models and descriptive statistics was 68,440. The sample was weighted using the survey weights provided by CCHS as instructed by Statistics Canada [30]. Descriptive statistics were conducted in SPSS Version 22.0 and regression modeling conducted using SAS 9.4 software. The alpha was set at 0.05.

### Dependent variables

The CCHS captured individual’s alcohol consumption through the original question “How often in the past 12 months have you had 5 or more drinks on one occasion?” R (18) [29]. As this is the only measure for alcohol consumption in the CCHS, we drew on the work of Thomas [19] and defined occasional any RSOD conservatively as having 5 or more drinks on one occasion in the past 12 months, infrequent RSOD as having 5 or more drinks on one occasion once or less than once a month and frequent RSOD as having 5 or more drinks on one occasion more than once a month [19]. In Canada, one standard drink contains approximately 14 g of pure alcohol, therefore RSOD in this case would be defined as having 70 g of alcohol in one sitting [24, 25]. Based on these definitions, we created two dependent variables:

1. RSOD which has two categories (binary outcome variable). ‘Yes’ if individual engaged in any RSOD in the past 12 months and ‘no’ if the individual did not engage in RSOD in the past 12 months.
2. Frequency of RSOD (multinomial outcome variable) which has three categories:
  i. No RSOD in the past 12 months
  ii. Infrequent RSOD: Over the past year, engaging in RSOD once a month or less
  iii. Frequent RSOD: Over the past year, engaging in RSOD more often than once a month

### Independent variables

Based on the relevant literature, we were able to identify independent variables likely associated with risky drinking, including age, sex, marital status, education status, income, employment, smoking status, self-perceived health, and self-perceived life stress [7, 31, 32]. As Thomas [19] identified, there is substantial provincial variation in alcohol consumption across Canada, and so we included the P/T variable as a geographic marker, as well as an indication of alcohol price and other regulatory policies. This comparison is possible as price (e.g., taxation) and regulation of alcohol differ by P/T. The P/T variable also stands to represent other historical, socio-political and cultural factors that may be present within jurisdictions. Age groups were categorized based on Thomas et al. [19]. Employment status was grouped as “employed last week” and “unemployed last week” to see the effect of employment status on RSOD, and was categorized based on the CCHS question, “Are you an employee or self-employed?” Total household income was captured and analyzed in increments of CAD 20,000 from <20,000 to 80,000 or greater. Education status was dichotomized to examine the significance of post-secondary education on RSOD. The highest level of education that is available from the CCHS survey data is post-secondary so we could not break it down further. Self-perceived life stress was assessed with the question “thinking about the amount of stress in your life, would you say that most days are: (not at all stressful, not very stressful, a bit stressful, quite a bit stressful, or extremely stressful)” and both responses quite stressful and extremely stressful were combined to increase the power of the model. Please refer to Table 2 for a full description of the frequencies associated with each dependent and independent variables.

**Table 2:** Distribution of the Canadian population according to independent and dependent variables, based on the CCHS cycle 2009/2010 (*n* = 68,440)

Variables	Freq (%)
Past 12 month RSOD	
None	48.4
Any	51.6
Past 12 month RSOD frequency	
Never	48.3
Infrequent	36.0
Frequent	15.6
Province	
Alberta	10.5
British Columbia	12.8
Manitoba	3.5
New Brunswick	2.3
Newfoundland & Labrador	1.7
Nova Scotia	2.9
Ontario	37.1
PEI	0.4
Quebec	25.8
Saskatchewan	2.8
Yukon, Northwest Territories & Nunavut	0.3
AGE	
12 to 17 years	2.9
18 to 24 years	10.7
25 to 39 years	28.1
40 to 59 years	42.3
60 years or older	16.0
Sex	
Male	52.2
Female	47.8
Marital status	
Single or never married	24.0
Common-law	13.7
Widowed, separated or divorced	10.3
Married	51.9
Type of smoker	
Daily	17.9
Always occasionally or occasionally	5.8
Former	42.2
Never smoked	34.1
Total household income (CAD)	
No or < $20,000	6.5
$20,000–$39,999	14.3
$40,000–$59,999	16.6
$60,000–$79,999	17.3
$80,000 or more	45.3
Perceived life stress	
Not stressed	9.6
Not very stressed	21.1
Bit stressed	43.6
Quite/Extremely stressed	25.3
Employment status	
Unemployed last week	25.8
Employed last week	74.2
Education	
Post-secondary	85.9
Less than post-secondary	14.1
Self-perceived health	
Excellent	24.3
Very good	39.9
Good	27.3
Fair or poor	8.5

### Analysis

We built two models: (1) a binary logistic regression model, and (2) a multinomial logistic regression model, to identify factors that were significantly associated with our dependent variables, any RSOD in the past 12months and frequency of RSOD in the past 12 months, respectively. We set the reference categories based on the group least likely to engage in RSOD according to the literature. We checked for absence of multicollinearity between the independent variables using Variance Inflation Factor (VIF) and found no issue (VIF less than 1.5). Backwards elimination method was used for modeling and all the variables were statistically significant.

## Results

### Descriptive statistics

After removing the cases with missing values for the dependent and independent variables from our sample, the sample size for both Model 1 and Model 2 was 68,440, equivalent to a complete response rate of 55.1% from the original survey sample size of 124,188. The estimated past-year prevalence of RSOD in the Canadian population is 51.6%. Further breakdown shows that 48.4, 36.0, and 15.6% of Canadians engage in no RSOD, infrequent RSOD and frequent RSOD, respectively. Therefore, among those who engage in RSOD, 70% do so infrequently while 30% do so frequently. For a full description of the frequencies associated with each dependent and independent variables, please refer to Table 2.

### Binary logistic regression (Model 1)

Please refer to Table 3 for a complete list of odds ratios and confidence intervals for each independent variable. After controlling for the other nine independent variables, we found that holding everything else constant, as an individual’s income level increased, the estimated odds for engaging in RSOD in the past year increased. In fact, the odds of the highest income group to indulge in RSOD were 1.77 times the odds of those in the lowest income group. The odds of males to engage in RSOD were 2.61 times the odds of females. Being married was significantly protective from engaging in RSOD, with the odds of RSOD being 1.77 times more in the single/never married category than in the married category. As individuals’ self-perceived health improved, and with an increase in smoking, an individual’s likelihood of engaging in RSOD also increased. Those who smoked daily had 3.63 times the odds of engaging in RSOD, than those who never smoked. The odds of RSOD among those who were employed last week were 1.32 times the odds of those who were unemployed last week. Individuals with less than post-secondary education had 1.09 times higher odds than individuals with post-secondary education. Finally, individuals in the adolescent (12–17) and young adult (18–24) age groups had much greater odds of engaging in RSOD than those aged 60 years or older.

**Table 3:** Odds ratios of variables associated with past-year RSOD (model 1) and odds ratios of variables associated with frequency of past-year RSOD (model 2) in Canada, based on the CCHS cycle 2009/2010

Model 1: logistic regression				Model 2: multinomial regression model					
RSOD vs No RSOD				Infrequent RSOD vs No RSOD			Frequent RSOD vs no RSOD		
Variables	OR	CI		RRR	CI		RRR	CI	
Province (Reference = Ontario)									
Alberta	1.16*	1.04	1.29	1.18*	1.05	1.32	1.08	0.92	1.26
British Columbia	1.03	0.93	1.14	1.05	0.94	1.17	0.96	0.83	1.11
Manitoba	1.29*	1.11	1.49	1.27*	1.09	1.48	1.39*	1.13	1.72
New Brunswick	1.63*	1.43	1.86	1.59*	1.38	1.84	1.79*	1.49	2.14
Newfoundland & Labrador	2.39*	2.05	2.79	2.14*	1.82	2.53	3.05*	2.48	3.75
Nova Scotia	1.71*	1.49	1.97	1.68*	1.45	1.95	1.79*	1.47	2.18
PEI	1.81*	1.49	2.20	1.86*	1.51	2.28	1.65*	1.23	2.20
Quebec	1.14*	1.05	1.25	1.20*	1.09	1.31	0.95	0.85	1.08
Saskatchewan	1.25*	1.10	1.42	1.25*	1.10	1.43	1.20	0.99	1.46
Yukon, Northwest Territories & Nunavut	1.65*	1.41	1.94	1.50*	1.27	1.77	2.03*	1.63	2.53
Age (Reference = 60 years or older)									
12 to 17 years	3.25*	2.74	3.85	3.42*	2.87	4.08	2.58*	2.14	3.56
18 to 24 years	8.09*	6.99	9.35	7.79*	6.70	9.05	9.62*	7.78	11.9
25 to 39 years	4.27*	3.87	4.71	4.25*	3.84	4.70	4.25*	3.57	5.06
40 to 59 years	2.03*	1.86	2.22	2.02*	1.84	2.22	2.16*	1.84	2.54
Sex (Reference = Female)									
Male	2.60*	2.44	2.77	2.32*	2.17	2.47	4.69*	4.23	5.21
Marital status (Reference = Married)									
Single or never married	1.71*	1.54	1.89	1.64*	1.47	1.82	2.12*	1.80	2.48
Common-law	1.51*	1.35	1.68	1.44*	1.28	1.61	1.93*	1.60	2.33
Widowed, separated or divorced	1.75*	1.60	1.91	1.59*	1.45	1.75	2.68*	2.35	3.07
Type of smoker (Reference = Never smoked)									
Daily	3.56*	3.22	3.93	3.16*	2.85	3.50	6.20*	5.29	7.26
Always occasionally or occasionally	3.41*	2.93	3.96	2.96*	2.54	3.46	6.35*	5.16	7.82
Former	2.29*	2.12	2.46	2.18*	2.02	2..35	3.01*	2.63	3.46
Income (Reference = No or < $20,000)									
$20,000–$39,999	1.04	0.90	1.21	1.09	0.93	1.28	0.96	0.79	1.25
$40,000–$59,999	1.13	0.97	1.32	1.15	0.98	1.35	1.10	0.90	1.36
$60,000–$79,999	1.38*	1.17	1.62	1.44*	1.21	1.71	1.28*	1.03	1.59
$80,000 or more	1.74*	1.49	2.03	1.79*	1.52	2.11	1.75*	1.42	2.16
Self-perceived life stress (Reference = Not stressed)									
Not very stressed	1.11	0.99	1.24	1.14*	1.01	1.28	0.99	0.85	1.16
Bit stressed	1.07	0.96	1.20	1.11*	1.01	1.25	0.90	0.77	1.04
Quite stressed	1.14*	1.01	1.29	1.16*	1.02	1.31	1.03	0.87	1.21
Employment (Reference = Unemployed)									
Employed	1.30*	1.20	1.41	1.31*	1.20	1.42	1.30*	1.14	1.49
Education (Reference = Post-secondary)									
Less than post-secondary	1.08	0.99	1.18	1.08	0.98	1.18	1.15*	1.03	1.28
Self-perceived health (Reference = Fair or poor)									
Excellent	1.35*	1.18	1.54	1.36*	1.19	1.56	1.30*	1.04	1.63
Very good	1.34*	1.19	1.51	1.37*	1.21	1.56	1.21	0.98	1.48
Good	1.18*	1.05	1.33	1.21*	1.06	1.37	1.09	0.89	1.34

* denotes statistical significance at 0.05

There was provincial variation in the RSOD of residents from different Canadian P/T. New Brunswick (NB), Nova Scotia (NS), Prince Edward Island (PEI), Newfoundland and Labrador (NFLD) and Yukon/North West Territories/Nunavut (YTN) had 1.64, 1.71, 1.80, 2.36 and 1.65 times the odds of engaging in RSOD than residents from Ontario, respectively. Therefore, individuals from the Maritime Provinces and Canadian northern territories had the highest of odds of engaging in RSOD compared to Ontario residents.

### Multinomial logistic regression (Model 2)

The multinomial logistic regression model (Model 2) shows the difference in the likelihood of those engaging in infrequent and frequent RSOD compared to those who ever engaged in any RSOD in the past 12 months. Many of the findings in Model 2 are consistent with Model 1. Similar to model 1, Individuals from Maritime provinces and northern territories consistently had higher odds of engaging in infrequent and frequent RSOD in the past year than residents of Ontario. One notable difference is that residents from Alberta, Quebec and Saskatchewan were found to be statistically no different than residents from Ontario when it comes to frequent RSOD, however residents in the above listed provinces are 1.18, 1.20, and 1.25 times more likely, respectively, to engage in infrequent RSOD than the residents of Ontario.

In terms of age, both Model 1 and Model 2 consistently show differences in the behavior of RSOD based on age group. For example, young adults aged 18 to 24 are 8 times and 10 times more likely to engage in infrequent and frequent RSOD than adults 60 years of age and older, respectively. Males dominantly engage in both infrequent and frequent RSOD compared to females. Consistent with the findings of model 1, being married is a protective factor against both frequent and infrequent RSOD. In addition, current and past smoking had a strong effect in engaging RSOD. For example, daily, occasional and former smokers are 6.85, 6.5, and 3.2 times more likely to engage in frequent RSOD than those who never smoke, respectively.

Perceived life stress is an interesting variable as it is associated with infrequent RSOD but not frequent RSOD. For example, people who perceive their life as quite or extremely stressful are 1.22 times more likely to engage in infrequent RSOD than those who perceive their life as not stressful at all; however, there is no difference between those who perceive their circumstances as quite stressful or extremely stressful versus non stressful when it comes to frequent RSOD. Therefore, stress is associated with infrequent RSOD but not frequent RSOD. In terms of income, Model 1 and Model 2 demonstrated similar directions of association wherein the two highest income groups are more likely to engage in RSOD than lowest income group holding everything else constant.

In terms of employment status, those who were employed in last week are 1.31 and 1.30 times more likely to engage in infrequent and frequent RSOD than those who were unemployed last week, respectively. Education is a protective factor for frequent RSOD but has no effect on infrequent RSOD. Excellent perceived health increase the likelihood of engaging in both infrequent and frequent RSOD. For example, those who identify as being in excellent health are 1.36 and 1.30 times more likely to engage in infrequent and frequent RSOD than those who identify as being in poor health, respectively.

## Discussion

This study is the first nation-wide study of factors, which are associated with the likelihood of individuals engaging in RSOD and frequency of engaging in RSOD in Canada. Multiple factors associated with RSOD identified in our models were consistent with the existing literature. Individuals who are male, unmarried or common-law, within the 18–24 year age range, and smoke were found to be more likely to engage in RSOD [1, 19, 31, 33] Other significant findings with regards to engaging in RSOD in the past year included: living in any province other than Ontario or British Columbia, being under 60 years of age, being widowed/separated/divorced, having a high income, reporting being stressed, being employed, and having a higher perceived health status. With regards to frequent RSOD, our findings were similar with notable differences identified for the P/T variable and the perceived life stress variable.

Socioeconomic status (SES), as a composite measure of an individual’s income, occupation, educational attainment, and social position in relation to others, has been generally linked to the risk of alcohol misuse including RSOD particularly among persons in the lowest SES strata [33–35]. Breaking down this measure into its principal components: income, occupation, and educational attainment, we find conflicting results between all three variables across both statistical models.

Our findings relating to educational attainment are consistent with the literature in which individuals with higher educational attainment drink less at an occasion than those with lower levels of educational attainment [34, 35]. An alternative hypothesis for this relationship, aside from SES, is higher educational attainment has been linked to an increased uptake of healthy behaviors including reduced smoking, increased physical exercise, and more moderate alcohol consumption [36, 37]. This further supports the role of education as a major determinant of health.

Individuals in the highest income brackets surprisingly had much higher odds of engaging in any RSOD, including both infrequent and frequent RSOD, compared to those in the lowest income bracket controlling for all other factors including education. This finding differs from the literature, as studies examining RSOD indicate that individuals with lower incomes and SES engage in drinking that exceeds low risk guidelines more often than their counterparts with higher incomes [33, 35]. The centers for disease control and prevention [38] surveillance of binge drinking (defined equivalently with RSOD in our study as 5+ drinks on a single occasion) has demonstrated that the prevalence of RSOD is highest among those with a high household income, however the intensity and frequency of RSOD is highest among those with the lowest household income [38]. Studies have also found that individuals with higher incomes tend to drink more often, however whether these individuals reach the threshold of RSOD is up for debate [34]. In addition, research on the relationship between alcohol use and wage earnings has demonstrated that individuals who drink earn higher wages than non-drinkers [39] however individuals who drink excessively earn less, suggesting an inverse u-shape relationship between alcohol use and wage earnings and income [40].

Currently there is limited Canadian literature on the relationship between employment factors and alcohol intake [41]. In this study, after controlling for all other independent variables, we found that being employed in the last week increased the odds of engaging in both infrequent and frequent RSOD compared to those unemployed. These findings, while inconsistent with the SES theory, are consistent with the findings of Marchand et al. [41] which found that hours worked per week were associated with increased frequency of RSOD. Moreover, Brown et al. [42] found unemployed individuals to be less likely to engage in heavy alcohol consumption, which the authors theorized could be due to a lack of funds or underreporting by this population group. This association between employment and RSOD may be further linked by type of employment and occupational status, unavailable in this dataset, and highlights an area for further exploration within the Canadian context.

As for the link between self-perceived health status and RSOD, we found in both models that as self-perceived health status is associated with an increase in the odds of infrequent and frequent RSOD. These findings differ from the existing literature outlining that increased alcohol use is correlated with lower self-perceived health scores [43]. However, studies have demonstrated that alcohol consumption decision making may be influenced by underlying health concerns [44]. It is possible that there may be a clinically-induced relationship between illness, pharmaceutical interventions, and the consumption of alcohol. This relationship was not explored in this study.

Interestingly, perceived stress increased the odds of infrequent RSOD but not frequent RSOD. Alcohol, as a depressant, is generally viewed as a coping mechanism for life stress and higher levels of stress in the form of job insecurity is linked to high-risk alcohol consumption [41]. However, one would expect that this relationship would extend into frequent RSOD.

We found that smokers, at any level, are much more likely to engage in RSOD and frequent RSOD compared to non-smokers. This relationship was much stronger at predicting frequent RSOD, suggesting a strong link between alcohol use and smoking. This finding is consistent with the literature linking smoking with alcohol abuse and other illicit drug use ([45, 46]).

As expected, marriage is associated with a protective effect on the risk of RSOD. However, persons living in common-law were more likely to engage in any RSOD and frequent RSOD than married individuals. This is interesting because common-law couples have enjoyed similar tax benefits and legal status in Canada as married couples due to high profile court cases Egan v. Canada [47] and M. v. H. [48]. Research on the differences between common-law and married couples has demonstrated that common-law couples are more likely to separate than married couples [49], are more likely to experience relationship strain [50], and enjoy fewer economic benefits [51]. The higher levels of instability in common-law relationships and households may contribute to the increased odds of RSOD and frequent RSOD.

Finally, province of residence was associated with the likelihood of engaging in RSOD. Persons living in Atlantic Canada or in one of the three Territories were more likely to engage in infrequent and frequent RSOD compared to Ontario. In the literature, comparative data pertaining to RSOD by province was not available; however, statistics show higher rates of overall consumption in the Territories, British Columbia, Alberta, and Newfoundland and Labrador [19]. This P/T variation is likely due to a myriad of factors including: alcohol policies and taxation, cultural norms, historical factors, and the social environment [19, 52, 53].

Although the legal age for purchase of alcohol in Canadian P/T is 19 years except in Alberta, Manitoba and Quebec, the regulatory system of alcohol sale is complicated. The retail liquor sale is wholly private (AB), exclusively controlled by Government (PEI, NB, NWT) or mixed controlled by both private and public systems (NS, QC, ON, SK, MB, BC, YT) [54]. The system is further complicated by permitting delisted products to be sold at significantly less than minimum price (SK), establishing special outlets for selling at discounted prices or allowing private liquor stores selling less than minimum price (BC) [55].

As for an association between P/T alcohol policies and RSOD, our findings did not uncover a link between the two variables. For instance, residents of Saskatchewan and Alberta had similar results with relation to RSOD; however these provinces employ very different alcohol policies. Saskatchewan has a mixed government and private sales structure whereby minimum prices are set in government stores and prices are indexed to inflation; whereas Alberta employs a free-market with regard to alcohol sales with privatization, no minimum prices, and no indexing to inflation [23]. Although factors such as price controls and tax policy have been shown to reduce regular alcohol consumption and heavy drinking [32], the effects of these forms of policy were not evident in our study. Therefore more direct research on this topic is needed to determine the best methods for provincial governments to control RSOD.

Our findings extend the existing literature by focusing on the Canadian population, and demonstrate that age, sex, education, income, employment, marital status, smoking status, life stress, self-perceived health, and province of residence are associated with RSOD and the frequency of RSOD.

### Limitations

There are some limitations to our study. The CCHS survey provides secondary data, which can restrict the availability and clarity of indicators pertaining to RSOD. For example, the provincial variable used in the research does not allow for interpretation of the factors within the province that may contribute to consumption (e.g., urban/rural, ethnicity). Moreover, using the provincial variable as a representation of the impact of alcohol policy proved challenging, as there too many confounding factors to draw a direct comparison. We could not explore the effects of different levels of post-secondary education using the “education” variable in the CCHS survey data because the variable had only one category for post-secondary education. The missing data also represent a possible bias in the results. We performed missing value analysis and determined that not all the missing data were MCAR. We attributed this to people who did not complete a large portion of the survey (failed to answer questions relating to both an independent and dependent variables).

At the same time, the range of definitions and terms (e.g., problematic drinking, binge drinking, risky drinking) used to define high risk alcohol consumption in Canada can make it difficult to directly compare and understand varying alcohol-specific research and policy. For example, the definition of risky drinking as employed by the CCHS survey may underrepresent the number of individuals engaging in risky drinking compared to the definition employed by the Canadian Centre for Substance Abuse (CCSA) as 4+ drinks for men and 3+ drinks for women, consumed on one occasion. Also, newer literature suggests a low-risk cut point of 10 g of pure alcohol per day, which is largely exceeded by what was captured by the CCHS survey (equivalent to approximately 70 g of pure alcohol). Finally, as reflected in the literature, the prevalence of health compromising behaviors such as alcohol overconsumption are likely underestimated in self-reported surveys, such as the CCHS [56].

## Conclusion and policy implications

This paper examined RSOD and frequent RSOD within the Canadian population. It was found that engaging in RSOD was significantly influenced by a myriad of demographic, socioeconomic, and health status variables. More research into the factors which we found to influence RSOD is needed to determine ways to mediate RSOD at all levels within the Canadian population as it is associated with negative health and social implications [7, 8].

It is important for policy-makers and researchers to start to consider the impact of people who participate in both infrequent and frequent RSOD, as those who engage in frequent RSOD are more likely to have serious negative health consequences however those who engage in infrequent RSOD remain at risk and represent a larger proportion of the Canadian population. As with Geoffrey Rose’s prevention paradox [57], targeting higher risk populations (frequent risky single occasion drinkers) for prevention is effective but not as effective as a mass prevention strategy in order to shift the whole population’s distribution of RSOD downwards.

Attempts at reducing RSOD through traditional health promotion efforts such as educational and media campaigns have proven largely ineffective [58], however studies have shown that changing perceptions about RSOD and cultural norms can be effective [59]. In addition research has shown that alcohol consumption is price-sensitive [60] and heavier drinkers may be more sensitive to price changes [61]. Therefore effective prevention strategies should include aspects of changing perceptions, especially among youth and young adults, about RSOD and cultural norms in conjunction with changes to provincial alcohol policies and taxation.

Additionally, our finding associating smoking with RSOD specifically suggests an opportunity to combine public health prevention strategies for both substances. Policies surrounding “cultural norms” with regards to RSOD, along with effective alcohol price controls are likely to reduce the prevalence and frequency of RSOD in Canada.

## Abbreviations

AB: Alberta
BC: British Columbia
CCHS: Canadian Community Health Survey
MB: Manitoba
MCAR: Missing completely at random
NB: New Brunswick
NL: Newfoundland and Labrador
NS: Nova Scotia
NT: Northwest Territories
NU: Nunavut
ON: Ontario
P/T: Provinces/Territories
PE: Prince Edward Island
QC: Quebec
RSOD: Risky single occasion drinking
SES: Socioeconomic status
SK: Saskatchewan
YT: Yukon
